# Socio-Economic Factors Affecting Member’s Satisfaction towards National Health Insurance: An Evidence from the Philippines

**DOI:** 10.3390/ijerph192215395

**Published:** 2022-11-21

**Authors:** Ardvin Kester S. Ong, Yogi Tri Prasetyo, Kate Nicole M. Tayao, Klint Allen Mariñas, Irene Dyah Ayuwati, Reny Nadlifatin, Satria Fadil Persada

**Affiliations:** 1 School of Industrial Engineering and Engineering & Management, Mapua University, 658 Muralla St., In-Tramuros, Manila 1102, Philippines; 2 International Program in Engineering for Bachelor, Yuan Ze University, 135 Yuan-Tung Road, Chung-Li 32003, Taiwan; 3 Department of Industrial Engineering and Management, Yuan Ze University, 135 Yuan-Tung Road, Chung-Li 32003, Taiwan; 4 Department of Industrial and Systems Engineering, Chung Yuan Christian University, Taoyuan 320, Taiwan; 5 Institut Teknologi Telkom Surabaya, Surabaya 60231, Indonesia; 6 Department of Information Systems, Institut Teknologi Sepuluh Nopember, Kampus ITS Sukolilo, Surabaya 60111, Indonesia; 7 Entrepreneurship Department, BINUS Business School Undergraduate Program, Bina Nusantara University, Jakarta 11480, Indonesia

**Keywords:** PhilHealth, SERVQUAL, ECT, structural equation modeling, deep learning neural network

## Abstract

The National Health Insurance, “PhilHealth”, is the healthcare provider for Filipino citizens in the Philippines. The study focused on determining the effects of members’ satisfaction with PhilHealth among Filipino members. The study utilized 10 latent variables from the integrated Service Quality (SERVQUAL) and Expectation-Confirmation Theory (ECT). There are 500 respondents that are used and analyzed through Structural Equation Modeling (SEM) and a Deep Learning Neural Network (DLNN). Utilizing SEM, it was revealed that Reliability, Responsiveness, Socio-Economic Factors, Expectation, Perceived Performance, Confirmation of Beliefs, and Members’ Satisfaction are significant factors in the satisfaction of PhilHealth members. Utilizing DLNN, it was found that Expectation (EX) is the most significant factor, and it is consistent with the results of the SEM. The government can use the findings of this study for the improvement of PhilHealth. The framework that is used for the analysis can be extended and can apply to future research with regard to its provided services. The overall results, framework, and concept utilized may be applied by other service industries worldwide.

## 1. Introduction

PhilHealth is the National Health Insurance provider in the Philippines. It was established under Act 7875 of 1995 (National Health Insurance Act) as an implementing agency with a mission to attain universal health insurance coverage to all Filipinos in 15 years’ time [1]. PhilHealth also acts as an instrument of the government that can provide equitable access to the highest possible quality of health services for Filipinos [1].

PhilHealth has introduced a primary care package that benefited millions of Filipinos covered under the program and expanded the benefit packages to include financial conditions. It implements a “no-balance billing” policy for the poor, so every Filipino can have access to quality healthcare [2]. PhilHealth’s top priorities are to provide for the healthcare needs of the underprivileged, the elderly, persons with disabilities, abandoned and neglected children, and workers who are not regularly employed in the informal sector.

PhilHealth’s primary mission is to ease the financial handicaps of members paying for their medical healthcare needs. Accredited PhilHealth hospitals, clinics, and laboratories are assured of their reimbursements incurred for the benefit of the confirmed PhilHealth member. As to the PhilHealth member, it assures them of discounts on their medical expenses. In a nutshell, PhilHealth is the third party between the PhilHealth member and the accredited medical institution rendering medical services to PhilHealth members, assuring that both parties receive the corresponding payment benefits. These benefits include inpatient benefits, outpatient benefits, Z benefits, and SDG-related benefits. Inpatient benefits are paid to the accredited Health Care Institution (HCI) through all case rates. The member’s total bill will deduct the case rate amount, including professional fees, before discharge. Outpatient benefits can cover the entire cost of day surgery, hemodialysis, radiotherapy, and other primary care. Z benefits are for the treatment for leukemia, prostate cancer, breast cancer, and cervical cancer. SDG-related HIV-AIDs and outpatient anti-tuberculosis treatment are also covered [2]. These aid in the satisfaction of PhilHealth users. Customer satisfaction plays a significant role in the success of the healthcare plan for Filipinos. Similarly, Thailand, Indonesia, Ghana, and Tanzania, also low and middle-income countries, have implemented the same programs as part of universal health coverage [3].

In 2017, PhilHealth earned a trust rating mainly attributed to efficiency in accrediting healthcare institutions, which improved access to PhilHealth services [2]. It earned a net satisfaction rating of over 92% from an individual customer [4]. The respondents claimed to have a satisfactory experience using PhilHealth [5]. PhilHealth’s consistent, reliable, and dynamic services were enough to earn a 95% trust grade for customer satisfaction and loyalty. Thus, the nation having PhilHealth as its medical arm for each citizen pursuant to its vision mission has been satisfactorily attained. In contrast, the survey conducted with Indonesian nurses shows an unsatisfactory result with the implementation of their National Health Insurance, which resulted in several challenges since the service systems implicate a discriminatory approach based on types of patients [6].

Past researchers have used various structures for satisfaction and reliability. The study of Capuno et al. [7] utilized Cox and Weibull’s Proportional Hazard Model to evaluate a similar arrangement of elements as leaned with outpatient and inpatients in the Philippines. Fulfillment Loyalty Theory with Expectation-Confirmation Theory [8] works on client devotion regarding public travel comprehension and gives a possible roadmap to future consumer loyalty. The review of Kersnik [9] utilized SEM to decide consumer satisfaction with an improved medical care framework, with the chance of free decision of family doctors and patient satisfaction employing postal overview. The past study integrates Ergonomics with the SERVQUAL model to produce a few examination apparatuses that can improve administration conveyance execution and assess the Philippine Government Agency in the Philippines [10]. According to the study of Johnson and Fornel [11], the Structural Equation Modeling (SEM) technique in the assessments of consumer loyalty ought to be founded on all the purchasing encounters of the client, ignoring a particular purchase insight. SEM can be used through personal information social occasions to maintain and further develop administration quality for administration patients and specialist co-ops through a multi-point-of-view structure [12]. Past studies also applied SERVQUAL, Expectation-Confirmation Theory, and Artificial Neural Network in customer satisfaction and loyalty.

The SERVQUAL model utilizes five dimensions: reliability, assurance, tangibles, empathy, and responsiveness [13]. It has been highly used to measure service quality to understand customer satisfaction [14]. In addition, service quality can be defined as “conformance to customer specification” [15]. The SERVQUAL method was utilized by [16] to evaluate customer experience to consider not only the point of expectation but the likelihood across the entire distribution of possible outcomes in customer satisfaction and loyalty. The study of Kottala [14] used SERVQUAL to establish patients’ insight, surpass assumptions while looking for treatment in the private well-being area, and connect quality measurements. Moreover, Sarreal [17] used the SERVQUAL method to establish clear linkages between customer satisfaction and the quality of their experience at the university. The SERVQUAL model is used to investigate the effect of service quality on satisfaction and identify the fulfillment of word-of-mouth correspondence in the general medical care industry [18]. Service quality of the healthcare sector, utilizing SERVQUAL, intends to distinguish the issue for future exploration on assistance quality in the medical care area [19].

Expectation-Confirmation Theory (ECT) is a model predicting and explaining satisfaction, customer loyalty, and continuance behavior [20,21]. User confirmation and satisfaction are the key predictors of satisfaction. Confirmation can express users’ expectations and lack of confirmation [22]. Leung and Chen [23] integrate ECT to investigate the prevalence and patterns of e-health/m-health that people engage via health-related technology. Concluding and forecasting whether patients they to utilize or mean to utilize cell phones to self-report clinical information operating ECT was considered by Reychav et al. [24]. Allowing the community to participate in the virtual community platform and to realize value by integrating ECT was also performed [25]. Different studies have utilized SEM and other tools to classify different factors affecting human behavior. Another advanced tool that can be utilized is the Deep Learning Neural Network (DLNN).

Deep Learning Neural Networks (DLNN) are a novel methodology currently receiving much attention [26,27]. DLNN describes a family of learning algorithms rather than a single method that can be used to learn complex prediction models, such as a multi-layer neural network with many hidden layers [28]. In the study of Emmert-Streib et al. [29], they utilized DLNN and were able to predict results in image analysis and speech recognition that have generated massive interest in many fields. Due to the heterogeneity of deep learning approaches, previous reviews are aimed at dedicated sub-topics [29]. A bird’s eye view without detailed explanation can be found in LeChun et al. [28]; a summary with detailed references can be found in Schmidhuber [30]; and reviews in the domains of image analysis and speech recognition can be found in [31,32]. DLNN can be used to predict patterns of emotions of the people in respective domain and the reason behind it was found to improve the customer experience and satisfaction [33]. Wickersham and McGee [34] integrated DLNN in an online course and found that even during deeper learning, the principles used have a positive result in the perception of satisfaction. Moreover, Rubin et al. [35] explored the interactive effect of age and gender in predicting surface and utilized DLNN in investigating these variables in relation to the degree of satisfaction. Several past studies have focused on assessing the members’ satisfaction through SERVQUAL. However, there is limited research regarding the National Health Insurance in the Philippines that measures member satisfaction towards its service. To address this gap, the researchers assessed the socioeconomic factors affecting members’ satisfaction through the integration of SERVQUAL, Expectation-Confirmation Theory, and a Deep Learning Neural Network.

This study aimed to identify factors that affected members’ satisfaction and to determine which factor will fall under the mandate of PhilHealth that can assess service qualities. Moreover, expectations that can apply to the National Health Insurance, “PhilHealth”, by integrating the SERVQUAL dimensions and Expectation-Confirmation Theory, assessed using SEM and Deep Learning Neural Network, will be applied. This study will be one of the first to assess satisfaction with using PhilHealth as insurance for healthcare in the Philippines. This study analyzed factors such as Reliability, Assurance, Tangibles, Empathy, Responsiveness, Perceived Performance, Expectation, Confirmation of Beliefs, Socio-Economic Factors, and Member Satisfaction. The findings will help to understand the stand of the members using PhilHealth and make it more functional for all the members. Results can improve services for a government agency realistically and make healthcare access available to all Filipinos. The analysis of this study can be the basis of research on customer satisfaction and customer loyalty among service utilities of a country. The framework and concept utilized may be applied by other service industries worldwide.

## 2. Methodology

### 2.1. Conceptual Framework

The variables that were integrated into the framework are SERVQUAL dimensions and Expectation-Confirmation Theory. Reliability, Assurance, Tangibles, Empathy, and Responsiveness come from the SERVQUAL Model, while Expectation, Perceived Performance, and Confirmation of Beliefs, come from the Expectation-Confirmation Theory. The remaining factors, such as Members Satisfaction and Socio-Economic Factors were added as an extension of the framework.

**Figure 1:**
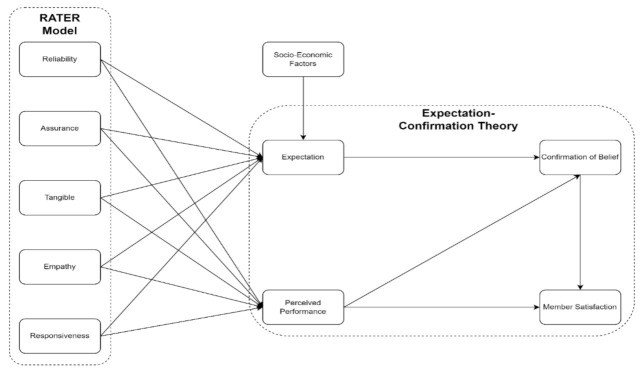
Conceptual Framework.

The conceptual framework in Figure 1 will assess PhilHealth members’ satisfaction by evaluating the members’ experience based on PhilHealth’s mandate, nature, and operations in the Philippines, which can satisfy and predict the members’ satisfaction using PhilHealth as an insurance provider. Quality can be a comparison between expectation and performance to measure how well the service level delivered matches the members’ expectations [36], wherein providing quality service means conforming to customer expectations consistently [37].

The variables under the SERVQUAL dimension are empathy, reliability, tangible, assurance, and responsiveness. In the framework, this can be an appropriate approach for assessing the quality of a firm’s service to measure consumer perception of quality. PhilHealth is one of the government agencies that can measure the service quality for members and accredited hospitals.

The variables under Expectation-Confirmation Theory (ECT) are expectation, perceived performance, and confirmation of beliefs. The ECT can be appropriate as a customer’s perception of how well their expectations, goals, and desires are being met [38]. PhilHealth members have only one desire: to have more significant discounts on their hospitalization expenses, especially on confinement, laboratories, physicians’ fees, etc.

Socio-Economic Factors also influence the expectation of the members; presently, unemployment is rampant, and COVID-19 entails financial expenses both from PhilHealth and its members.

Regarding interactions between the SERVQUAL and ECT in Figure 1, it will help in facilitating the aspect of service quality and expectations of PhilHealth members. The researchers will investigate how service quality and expectations can influence members’ satisfaction through the conceptual framework. Previous studies have indicated that the service quality dimension mostly affects customer satisfaction, and ECT can express users’ expectations and lack of confirmation [14,39].

**Figure 2:**
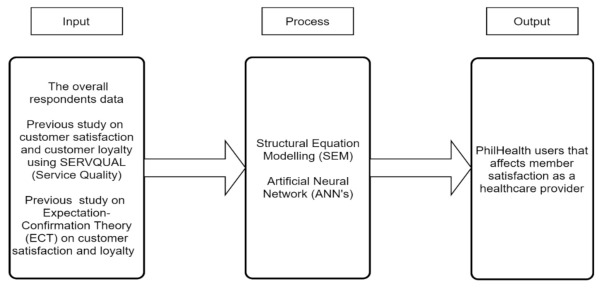
Input–Process–Output Framework.

The input–process–output model in Figure 2 can be used in system approach analysis, and software that can identify the input, output, and the required processing tasks is required to transform input into outputs. Hence, it is logical to base this study on facts or theories regarding satisfaction and expectation.

SERVQUAL, as a prediction of service quality, has been considered an ideal standard [40]. Berry et al. [41] stated that the service quality assessment results from customers comparing their service quality expectations to their perceived service has a positive effect. According to Saleh and Ryan [42], past customer experience plays a role in the formulation of expectations. The expectations of customers these days consistently change direction, grow, shrink, change shape, and adapt to the environment. Their demands, needs, and wants will decide how they feel about the level of service and their satisfaction, which will be dictated by how well the company meets their expectation using the Rater Model. Thus, it was hypothesized that:

**Hypothesis** **1** **(H1).** Reliability has a positive relationship with Expectation;

**Hypothesis** **2** **(H2).** Assurance has a positive relationship with Expectation;

**Hypothesis** **3** **(H3).** Tangible has a positive relationship with Expectation;

**Hypothesis** **4** **(H4).** Empathy has a positive relationship with Expectation;

**Hypothesis** **5** **(H5).** Responsiveness has a positive relationship with Expectation.

Parasuraman et al. [13] conceptualized reliability as the “ability to perform and provide service dependability and accurately”. Zeithaml and Bitner [43] argue that service quality can play out the guaranteed benefit reliably and precisely. For the customer, the important thing is what the firm promises and whether or not it delivers on those promises [44]. Parasuraman et al. [13] referred to confirmation as “employees” knowledge, courtesy, and ability to inspire and create trust and confidence. The things that have a physical existence can be seen or touched are called tangibles; these tangibles are randomly integrated by any organization to render services to its customers [45]. Individuals’ form perceptions of existing services based on the treatment they receive and the empathy dimensions of service providers, including the ability to understand the individual’s necessities [43,45]. In addition, reliability has a positive relationship with Perceived Performance. Therefore, it was hypothesized that:

**Hypothesis** **6** **(H6).** Reliability has a positive relationship with Perceived Performance;

**Hypothesis** **7** **(H7).** Assurance has a positive relationship with Perceived Performance;

**Hypothesis** **8** **(H8).** Tangible has a positive relationship with Perceived Performance;

**Hypothesis** **9** **(H9).** Empathy has a positive relationship with Perceived Performance;

**Hypothesis** **10** **(H10).** Responsiveness has a positive relationship with Perceived Performance.

Socio-Economic factors have triggered several unprecedented changes, e.g., the uncertainty related to public safety that can impact individuals, mental health, and depression. It also influences public awareness [46]. With that, the expectation of individuals may be preceded by their socio-economic status. Liu et al. [47] stated that there is positive or negative disconfirmation of beliefs with customer expectations through the product or service performance. As to the users’ accumulated experience from the system usage, their thoughts and attitudes may change. Contrary to customers’ expectations, customer evaluation of performance should affect the perception of expectancy disconfirmation [47]. Thus, it was hypothesized that:

**Hypothesis** **11** **(H11).** Socio-Economic factors have a positive relationship with Expectation;

**Hypothesis** **12** **(H12).** Expectation has a positive relationship in Confirmation of Beliefs.

Socio-Economic triggered several unprecedented changes, the uncertainty related to public safety that can impact individuals, mental health, and depression. It also influences public awareness [46]. With that, the expectation of individuals may be preceded by their socio-economic status. To which, Liu et al. [47] stated that there is positive or negative disconfirmation of beliefs with customer expectation through the product or service performance. As to the users’ accumulated experience from the system usage, thoughts and attitudes may change. Contrary to customers’ expectations, customer evaluation of the performance should affect the perception of expectancy disconfirmation [47]. Thus, it was hypothesized that:

**Hypothesis** **13** **(H13).** Perceived performance has a positive relationship with Confirmation of Beliefs;

**Hypothesis** **14** **(H14).** Perceived performance has a positive relationship with Members Satisfaction;

**Hypothesis** **15** **(H15).** Confirmation of Beliefs has a positive relationship with Customer Satisfaction.

### 2.2. Structural Equation Modeling

Using Structural Equation Modelling (SEM) allows on to evaluate the relationship between the factors and the latent variables affecting members’ satisfaction using PhilHealth. SEM is a multivariate statistical method that defines observable and unobservable variables in a model based on a specific theory [48]. SPSS 25 and AMOS 25 were utilized to run the SEM. According to Savari et al. [49] one of the main purposes of using SEM in research is because it is a convenient approach to evaluate the theory of the research. Furthermore, it is suitable to evaluate the exogenous and endogenous latent variables to determine the compatibility of the results [50].

**Figure 3:**
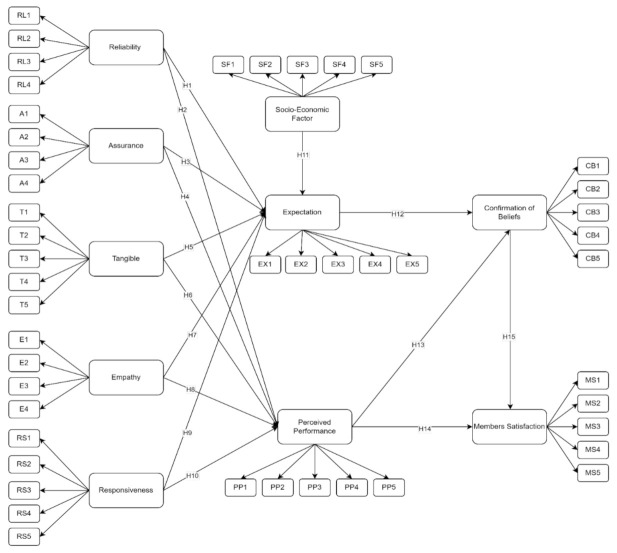
SEM Model Framework.

The initial SEM in Figure 3 consists of latent variables and indicators. The model itself has 10 latent variables with 2 exogenous latent variables (Member Satisfaction and Socio-Economic Factors) and 8 endogenous latent variables (Reliability, Assurance, Tangible, Empathy, Responsiveness, Perceived Performance, Expectation, and Confirmation of Beliefs). For the SERVQUAL model, the latent variable will have 22 indicators, and the rest of the latent variables contain 5 indicators each.

### 2.3. Respondents

This study has a total of at least 500 respondents whom responded to an online survey (Table 1). The online survey was dispersed using Google Forms to all Filipinos that are residing in the Philippines who are members of PhilHealth. Using convenience sampling, the questionnaire was distributed online due to the COVID-19 pandemic. Following the study of Ong et al. [50], as long as at least 500 respondents were gathered, the SEM and their constructs would be generalizable for analysis and interpretation [51].

### 2.4. Questionnaires

The respondent’s survey was measured using a 5-point Likert scale. The questionnaire (Table 2) had the options of Strongly Disagree (1), Disagree, Neither Agree nor Disagree, Agree, and Strongly Agree (5). The questionnaire consisted of 3 sections: (1) Demographics (Age, Gender, Income/Allowance, Member of PhilHealth, Province/City of Residence, Years member of PhilHealth); (2) Service Quality (Reliability, Assurance, Tangibles, Empathy, and Responsiveness); (3) Expectation-Confirmation Theory (Perceive Performance, Expectation, Confirmation of Beliefs, and Member satisfaction); and (4) Socio-Economic Factors.

### 2.5. Deep Learning Neural Network

The collected data for this study were from 500 Filipino respondents, which presented a total of 23,500 datasets (500 × 47) for predicting factors affecting the members’ satisfaction with PhilHealth. The only response from the Likert Scale survey was considered in this study following the study of Ong et al. [71]. In accordance, Yuduang et al. [72] utilized a similar methodology following a data pre-processing stage. The collected data show that most females (52.8%) and males (46.8%) ranging from 18 to 75 years old are all collected around NCR. The majority of the respondents are members of PhilHealth (95.6%). Hence, most of the respondents have already been a member of PhilHealth for 1 to 10 years (42.8%), with monthly salaries/allowances over PHP 15,000–30,000 (40%), above PHP 30,000–45,000 (28.2%), over PHP 45,000–60,000 (13.6%), and above PHP 75,000–90,000 (11.2%).

Data preparation was performed before running the MLA, adopted from different studies [71,72]. This study utilized SPSS 25 to review the data and found that there were no missing data, and the data were pre-processed. Moreover, correlation is also used to clean data to isolate the non-significant indicators and remove them from the optimized data. It was seen that indicators with a *p*-value of less than 0.05 are significant, and a correlation greater than 0.20 is considered for MLA-optimization. All indicators were seen to be significant. Data aggregation was used to summarize the data by obtaining the average of the different indicators for the MLA optimization. Hence, nine latent variables were considered (RL, A, T, E, RS, PP, EX, CB, and SF). Data normalization was performed utilizing Python 3.8. Initial optimization was performed using a deep learning neural network after the data normalization to predict the factors affecting the member’s satisfaction of PhilHealth members. This process implicated how the responses would be linked to members’ satisfaction with the national health insurance. Similar to the study of Yuduang et al. [72], SEM and neural network analysis were conducted to verify the findings and close the limitations brought by SEM [49].

Deep Learning Neural Network (DLNN) is a type of Neural Network (NN) used due to its great ability to generalize and classify data [71,72]. DLNN can mimic the structures of the human brain and central nervous system to recognize hidden patterns among datasets [72]. It has at least two hidden layers of patterns, containing an input layer and an output layer responsible for prediction [71]. Furthermore, DLNN has also demonstrated excellent performance in many classification-related medical imaging applications [73]. DLNN can sufficiently simulate the distribution of the input data due to a large number of parameters [74]. Various studies used DLNN with SEM [75,76,77], which can offer appealing benefits and together serve as a suitable tool for the study. Hence, in this study, there are 9 nodes (RL, A, T, E, RS, PP, EX, CB, and SF) that are utilized for the input layer in predicting members’ satisfaction using PhilHealth in the Philippines.

The study used various combination of activation functions (AF) and optimizers summarized in Table 3. Using different AF and optimizer, there are four AFs for the hidden layer that were considered: Swish [78,79,80], Tanh [81,82,83], Elu [84,85,86], and Sigmoid [87,88,89]. Two AFs are considered for the output layer: Sigmoid [79,81,88,89] and Softmax [90,91]. Lastly, three optimizers are used, namely, Adam [92,93,94], RMSProp [90,95,96], and SGD [97,98].

## 3. Results and Discussion

### 3.1. Structural Equation Modeling Results

**Figure 4:**
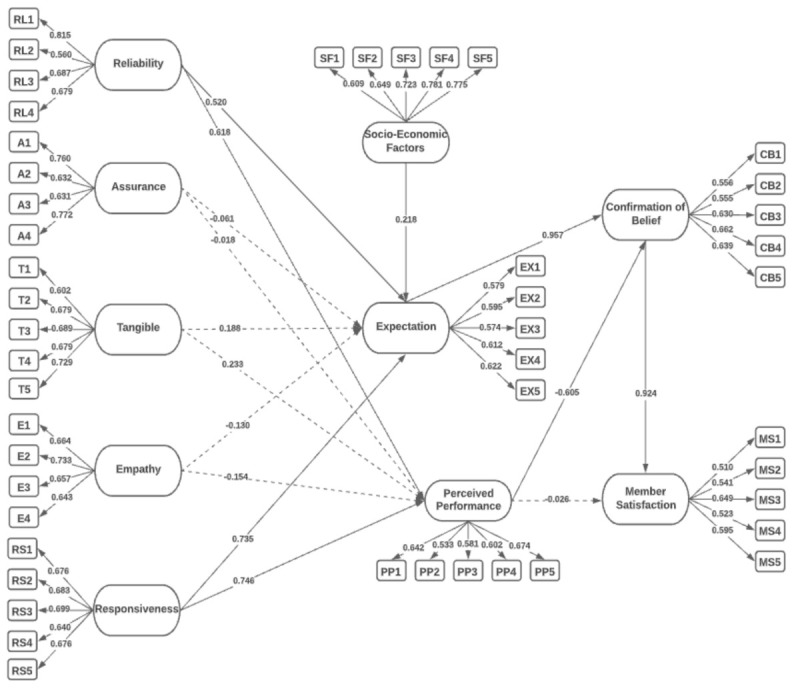
Initial SEM Model for determining the Members’ Satisfaction towards PhilHealth.

**Figure 5:**
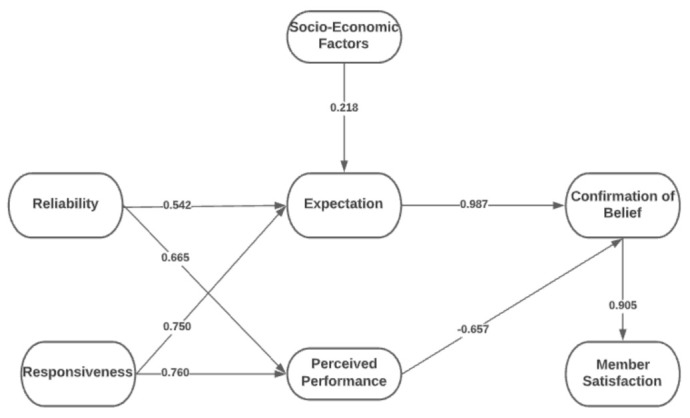
Final SEM Model of determining the Members’ Satisfaction towards PhilHealth.

Figure 4 demonstrates the initial model for determining the factors that affect the members’ satisfaction with the National Health Insurance, “PhilHealth”, among Filipino members. The initial SEM model was reconstructed to strengthen the model’s fit by removing non-significant latent (*p*-value > 0.050) indicators having values less than 0.50 [51]. It was found that 3 out of 10 hypotheses are not significant factors. Hence, a revised SEM was derived by removing these hypotheses. Figure 5 demonstrates the final SEM model for determining the factor that affects the members’ satisfaction with PhilHealth.

Table 4 presents the descriptive statistics of the factor loading of the initial and the final SEM model for determining the factors that affect the members’ satisfaction among the Filipino members of PhilHealth. Table 4 presents the initial and final analysis of the indicators that are presented by the study.

The model fit is presented in Table 5. The IFI, TLI, CFI, GFI, and AGFI are higher than the suggested cut-off of 0.80 [100], indicating that the model’s hypothesized construct was past the presentation of the observed data. The Root Mean Square Error (RMSEA) with a value less than 0.07 is also acceptable and fit reasonably [101]. Thus, the model is said to be acceptable [102].

Table 6 presents the reliability and validity of the constructs. The Average Variance Extracted (AVE) can measure the validity of all indicators for the final model. According to Knekta et al. [103], factor loading has a minimum cut-off of 0.50. Moreover, AVE also has a minimum cut-off of 0.50. In the study of Pervan et al. [104], if the value of Composite Reliability (CR) is greater than 0.60, then the construct validity is considered accepted. Moreover, Ong et al. [50] explained that Cronbach α and Composite Reliability (CR) can measure internal consistency, reflects how reliable the items used to reflect constructs are when the value is greater than 0.7. Thus, the overall constructs demonstrate good values for Cronbach’s α, AVE, and CR.

Table 7 represents the direct, indirect, and total effects of different indicators. The SEM model was performed in AMOS 25 and SPSS software to gather the results. Based on the model, four indicators significantly affect the Expectation of users towards the Confirmation of Beliefs (CB), which are Socio-Economic Factors (SF), Responsiveness (RS), and Reliability (RL). At the same time, Empathy (E), Tangibles, and Assurance (A) have no significant effect on the Expectation. However, three indicators have a substantial impact on the Perceived Performance, which are Responsiveness (RS), Tangibles (T), and Reliability (RL). Furthermore, Confirmation of Beliefs (CB) has a substantial impact on the Member Satisfaction (MS) of PhilHealth users.

### 3.2. Deep Learning Neural Network Results

The initial optimization was executed by performing 10 runs for each combination with 150 epochs [105]. Furthermore, the number of nodes for the hidden layer was optimized by increments of 10 until reaching 100. Thus, the number of overall runs for the initial optimization of the data considered was 21,600 runs. The summary of the initial DLNN optimization is presented in Table 8.

The study performed an ANOVA for the initial run to determine the most significant latent variable. Based on the results, Expectation (EX) had an average accuracy of 85.67% with a standard deviation of 2.946. Second to highest was Empathy (E), which had an average accuracy of 81.80% with a standard deviation of 3.155, followed by Perceived Performance (PP) and Assurance (A), having an average result of 80.70% and standard deviations of 3.129 and 3.335, respectively. The parameters that are used for EX were Tanh as the AF hidden layer, Sigmoid as the AF output layer, and Adam as the optimizer for the final optimization.

**Figure 6:**
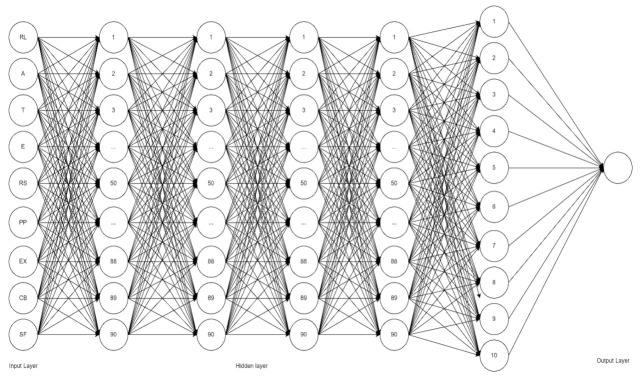
Deep Learning Neural Network.

For the final optimization, the best average accuracy was found by running 4 hidden layers with 200 epochs using the combination of Tanh as the AF hidden layer, Sigmoid as the AF output layer, and Adam as the optimizer. Training and testing ratios of 80:20 and 70:30 were used. We used Tanh as the AF of the hidden layer, Sigmoid as the AF of the output later, and Adam as the optimizer for the final DLNN, which showed an average accuracy of 90% with a 4.46 standard deviation at an 80:20 training:testing ratio. Expectation (EX) was found to be the most significant factor that affects the members’ satisfaction, which is consistent with the results of the SEM. Presented in Figure 6 is the DLNN for predicting factors affecting members’ satisfaction using PhilHealth in the Philippines.

### 3.3. Discussion

TSEM and DLNN were utilized in this study to identify factors that can affect members’ satisfaction with PhilHealth and to determine which of the factors fall under the mandate of PhilHealth. In using SEM, it was found that EX has the highest significant factor that affects the members’ satisfaction (β = 0.987, *p* = 0.004). In addition, DLNN resulted in an average accuracy of 90% for EX, the highest factor influencing customer satisfaction. According to the mandate of PhilHealth, it indicates that the qualifications and capabilities of healthcare are to assure that the health services meet the desired and expected quality [2]. Therefore, EX is one of the significant factors that can affect the members’ satisfaction utilizing SEM and DLNN.

The indicators of EX that affected CB include lessened hospital bills, providing healthcare suggestions, ease of transactions, prompt services, and accurate information. Based on the study of Casad [106], beliefs can include one’s expectations in a situation and can predict its outcome. Moreover, people are likely to process information to support their own opinions when the issue is fundamental [106]. Hence, based on the results, PhilHealth members have already experienced such hospital bill discounts and good medical care services from hospitals and thus exceed their beliefs in PhilHealth services.

The second most significant direct effect was CB on MS (β = 0.905, *p* = 0.028). The indicators that affected MS include the expected quality, providing more information, benefiting from healthcare, gaining quality service, and expectation of users. Beliefs relate to emotional outcomes, especially in satisfaction. Improving services would directly affect the satisfaction of the users [107]. Consumer satisfaction during COVID-19 was impacted by employees’ norm-conforming and norm-violating behaviors related to COVID-19 [108]. Based on the results, PhilHealth provides the best for PhilHealth members, which satisfied their needs in healthcare. Furthermore, based on the DLNN results, CB was also seen to have a highly significant factor, with 81.50% average accuracy.

Third, RS was seen to have a significant effect on PP (β = 0.760, *p* = 0.008) and EX (β = 0.750, *p* = 0.012). Based on the results, PhilHealth is responsive and able to meet the members’ expectations, especially in financial matters and medical services. They emphasized how people-centered a health system is and to what extent the legitimate expectation of the members is met. Quality of healthcare depends not only on the effectiveness and the medical aspects of care but also on the interface between the health services and communities [109].

Fourth, RL has a significant effect on PP (β = 0.665, *p* = 0.007) and on EX (β = 0.542, *p* = 0.006). Reliability is one of the most essential factors in customers’ or users’ judgments of the performance and expectations of a service [110]. Most individuals still focus on the economy and ignore the factor validity and performance reliability of human cognitive behavior, which can impact the environment [111]. Based on the results, PhilHealth members have declared their satisfaction with regard to the proposed financial discounts offered to members as well as good medical care and services. This justifies the indicators presented under RL. PhilHealth members believed that the accredited healthcare institutions are knowledgeable, execute good services, are accommodating, and have good performance.

Fifth, SFs have a significant effect on EX (β = 0.218, *p* = 0.008). It is of great significance to premium members to avail the benefits of PhilHealth, including (1) cheaper hospital bill payables and (2) considerate hospital services and facilities. According to Adler and Newman [112] and the American Psychological Association [113], health insurance members will require a policy initiative addressing socioeconomic components [114]. In the study of Kurata et al. [115], only 47% indicated that socio-economic factors influence their use and satisfaction with the healthcare provider. PhilHealth members indicated that they are satisfied with the discount, the accredited healthcare providers, and the organizational charts, services, and contributions.

Surprisingly, PPs have a negative, significant, direct effect on CB (β = −0.657, *p* = 0.028). In the results in the model, PhilHealth in general might have felt the needs of members for their welfare. Perhaps members made numerous complaints regarding the discounts and services of PhilHealth. Based on the indicators, it could be deduced that people still perceive it as costly, the quality in general may be questionable, incentives are not reasonable, there is a lack of responsiveness among accredited professionals in the accredited hospitals, and claim settlement may not always occur on time. In the study of Isac [116], the discrepancy between a pre-purchasing standard (expectation or desires) and actual performance was seen to have a negative effect, as well [117]. This justifies that E, T, and A were not considered significant. The indicators provided from PP affect the feelings of people towards the emotional performance of the professionals when utilizing the PhilHealth membership. Due to the availability of services from PhilHealth, professionals are providing their services but may lack empathy. Thus, people do not feel assured when consulting healthcare professionals or when they need assistance. Meesala and Paul [118] indicated that A, E, and T were not significant when it comes to patient satisfaction in India. They indicated that satisfaction would only be considered by loyal customers of the hospitals.

The results utilizing the SEM and DLNN would be a significant help to the National Health Insurance “PhilHealth” because it brings awareness to the factors that can affect the members’ satisfaction. This study found factors with a significant effect on the members’ satisfaction within the mandate of PhilHealth that the government can improve to make Filipinos satisfied with the service of PhilHealth. Thus, the findings of this study can be a great contribution to the government to improve the service of PhilHealth and satisfy the needs of all Filipinos.

### 3.4. Theoretical Implications

SERVQUAL and ECT, analyzed using SEM and DLNN, showed that expectation has the highest results. Expectation can be a beneficial factor to evaluate the customer experience that can lead to customer satisfaction [16]. The results implied that members often expect great services from the utilities provided to them, which affects the overall members’ satisfaction. In the study of Leung and Chen [23], expectation can predict and explain satisfaction in terms of members’ satisfaction.

The integrated theories, SERVQUAL and ECT, could be utilized to contribute to customer satisfaction towards the provided services. The SERVQUAL model aids in assessing the quality of a firm’s service to measure the consumer perception of quality [41]. Several factors of SERVQUAL (RL and RS) were found to have a direct significant relationship with EX and PP in ECT. The ECT can be an appropriate tool for assessing customers’ perceptions and expectations [38]. It was revealed that CB has the second most significant relationship with members’ satisfaction after EX. Therefore, integrating both SERVQUAL and ECT can be great model to be utilized in holistically determining the factors that affect the members’ satisfaction. The integrated models can be utilized and extended to further studies to determine the members’ satisfaction of provided services in various fields such as firms, the automotive industry, food delivery, and marketing.

This contribution also aimed to provide originality surrounding the factors that affect the members’ satisfaction, especially in terms of healthcare during the new normal of COVID-19. Moreover, PhilHealth has a big contribution to the new normal of COVID-19. There were additional factors that members needed in healthcare, and these factors were modeled and analyzed using the SEM and DLNN to determine factors that affect members’ satisfaction. SEM and DLNN can recognize the relationship between the constructed variables and can predict the output significantly more accurately. Thus, this can justify that the result from the combined SEM and DLNN can be utilized for human behavior and measuring customer satisfaction.

### 3.5. Practical Implications

The findings of this study suggest that PhilHealth must focus on responsiveness, perceived performance, assurance, and empathy in delivering high-quality services to achieve high satisfaction from its members. The management of PhilHealth must continue to provide employee training, particularly on service, to enhance their skills. This will help the firm deliver fast and reliable service to all its members. The findings can also identify and help understand the members’ needs and expectations.

The results stated that 95.60% of respondents are members of PhilHealth; they are all expecting a good quality service that PhilHealth can provide to all Filipino members. The government can implement a better service for all PhilHealth members to satisfy their needs as Filipino citizens. Considering PhilHealth’s current situation, it could be seen that the mission has not been realistically carried out, mainly in terms of the much-expected members’ benefits. As stated in the results utilizing SEM and DLNN, expectation is the most significant factor affecting members’ satisfaction. Thus, PhilHealth members’ expectations include many benefits for their own welfare, especially regarding hospitalization, confinement benefits, as well as for laboratories. Therefore, focusing on improving these benefits by reducing the percentage of the members can be more helpful to all Filipinos.

Given the above, both employees and the government can revamp the management policies and future member benefits that the Filipinos can rely on. It can be suggested to use “kaizen” activities by turning member feedback and concerns into positive inputs to improve service quality [54]. These improvements can be beneficial to the members. Hence, evidence has been provided that a better quality of services will significantly improve satisfaction [54].

### 3.6. Limitations and Future Research

There are some limitations that need to be considered in this study. This study utilized the SERVQUAL and ECT theories. However, it only evaluated responses from Filipino respondents. To further highlight the applicability and holistic measurement of customer satisfaction, the model may be applied and extended in other service industries and in other developing countries. Moreover, the data were gathered through an online self-administered survey, and this study was only able to measure member satisfaction due to COVID-19. Employing interviews may be beneficial to determine other factors that may contribute to customer satisfaction. There are still factors and tools that can consider using this type of study, thus limiting its findings. Future research can apply and extend the models that were utilized in this study to determine customer satisfaction with regards to provided services.

## 4. Conclusions

PhilHealth is the National Health Insurance in the Philippines, handled by the government. Members’ satisfaction is commonly studied in every country to identify the service quality of a firm or company. Hence, the researchers decided to study the factors that affect PhilHealth members’ satisfaction with the service by incorporating factors from SERVQUAL and Expectation-Confirmation Theory (ECT) using Structural Equation Modeling (SEM) and a Deep Leaning Neural Network (DLNN). In utilizing the SEM and DLNN, the highest significant factor for member satisfaction was Expectation (EX). Based on the SEM results, Reliability, Responsiveness, Socio-Economic Factors, Expectation, Perceived Performance, Confirmation of Beliefs, and Members’ Satisfaction are the significant factors among Filipino members affecting members’ satisfaction using PhilHealth. This study is also one of the first studies that utilized SERVQUAL, ECT, and DLNN in determining factors affecting members’ satisfaction towards PhilHealth. It was found that several factors of SERVQUAL (RL and RS) have a direct significant factor to ECT (EX and PP).

The overall study determined that the factors that affect the members’ satisfaction with PhilHealth are RL, A, T, E, RS, PP, EX, CB, SF, and MS. EX was determined to be the most significant factor for members’ satisfaction towards PhilHealth among the Filipino members. PhilHealth members expect to have lower hospital bills and to receive better benefits compared to non-PhilHealth members since a high percentage of their salary is removed to contribute to PhilHealth. Therefore, the study suggests providing employee training and revamping management policies for better services for Filipino members under PhilHealth. Moreover, the current research can realistically improve services for a government agency and make healthcare access available to all Filipinos. The results of this study can be the basis of research on customer satisfaction and customer loyalty among service utilities in the country. Furthermore, the government should improve the mandate of PhilHealth, wherein most of the members will benefit from much better healthcare and so that PhilHealth can meet the expectations of the members. This study contributed to identifying the members’ satisfaction towards PhilHealth utilizing SERVQUAL and ECT in a framework. Future research can apply and extend the models that are utilized in this study to determine the customer satisfaction with regard to its provided services worldwide.

## Figures and Tables

**Table 1:** Demographics.

Characteristics	Category	*n* = 500	Percentage
Age	18 to 25	135	27%
	25 to 45	186	37%
	45 to 60	122	24%
	60 to75	57	11%
Gender	Female	264	53%
	Male	234	47%
	Others	2	0%
Income/Allowance	Below 15,000	56	11%
	15,000–30,000	200	40%
	30,000–45,000	141	28%
	45,000–60,000	68	14%
	60,000–75,000	12	2%
	75,000–90,000	23	5%
Member of PhilHealth	Yes	478	96%
	No	22	4%
Years Member of PhilHealth	0	18	4%
	1–10 years	214	43%
	10–20 years	128	26%
	20–30 years	99	20%
	30–40 years	35	7%
	40–50 years	6	1%
	50–60 years	0	0%
Province	Manila	273	55%
	Laguna	52	10%
	Makati	44	9%
	Las Pinas	29	6%
	Cavite	8	2%
	Paranaque	5	1%
	Bohol	1	0%
	Malabon	1	0%
	Samar	1	0%
	Taguig	12	2%
	San Juan	9	2%
	Pagsanjan	1	0%
	Muntinlupa	25	5%
	Pasay	11	2%
	Pangasinan	1	0%
	Bulacan	6	1%
	Cebu	1	0%
	Rizal	6	1%
	Pampanga	8	2%
	Romblon	1	0%
	Caloocan	1	0%
	Q.C	1	0%
	Tarlac	1	0%
	Batangas	2	0%

**Table 2:** Questionnaire.

Construct	Items	Measures	References
Reliability	RL1	Accredited hospital staffs are knowledgeable about their job.	Devicais [52]
	RL2	Accredited hospitals execute good services.	Goula et al. [53]
	RL3	Accredited hospitals provide accommodation for walk-in patients.	Balinado et al. [54]
	RL4	Accredited hospitals provide performance launched by staff and doctors.	Chang et al. [55]
Assurance	A1	Accredited hospital staff responds to every patient.	Kitapci et al. [18]
	A2	Accredited hospitals allow patients to ask questions.	Devicais [52]
	A3	The hospital staff can accommodate PhilHealth users	Goula et al. [53]
	A4	Accredited hospitals inform patients when they are next in line.	Balinado et al. [54]
Tangibles	T1	Accredited hospitals provide well-ordered polyclinic service.	Kitapci et al. [18]
	T2	Accredited hospitals provide a clean and maintained bathroom.	Chang et al. [55]
	T3	Accredited hospital staff are dress properly and appear neat.	Goula et al., [53]
	T4	Accredited hospitals have convenient consultation hours.	Kitapci et al. [18]
	T5	Accredited hospitals provide a comfortable waiting area for patients.	Devicais [52]
Empathy	E1	Accredited hospital staff apologizes when committing mistakes.	Balinado et al. [54]
	E2	Accredited hospital staff is courteous and considerate in dealing with patients.	Devicais [52]
	E3	Accredited hospital staff understands the specific needs of patients.	Kitapci et al. [18]
	E4	Accredited hospital staff assists the patient in a caring manner.	Balinado et al. [54]
Responsiveness	RS1	Accredited hospital staff responds to service inquiries.	Balinado et al. [54]
	RS2	Accredited hospitals provide prompt yet accurate service.	Devicais [52]
	RS3	Accredited hospitals provide interest in solving a problem.	Kitapci et al. [18]
	RS4	Accredited hospitals provide a willingness to help a patient in a good response capacity.	Hernández-Gracia et al. [56]
	RS5	Accredited hospital staff has the confidence to answer the question.	Hernández-Gracia et al. [56]
Perceived Performance	PP1	The accredited hospital provides a perception of service quality concerning cost.	Fu et al. [8]
	PP2	The accredited hospital provides good service quality.	Fu et al. [8]
	PP3	The accredited hospital provides reasonable incentives for every Filipino.	Health & Corporation [57]
	PP4	The accredited hospital is responsive and professional in terms of medical care	Health & Corporation [57]
	PP5	The accredited hospital provides claim settlement on time.	Koopmans et al. [58]
Expectation	EX1	The accredited hospital can lessen hospital bills.	Abasi et al. [59]
	EX2	The accredited hospital provides suggestions about health care.	Waraporn Sirithammanuku, BNS et al. [60]
	EX3	The accredited hospital provides ease in obtaining follow-up information and care (test results, medicine).	Srivastava & Goel [61]
	EX4	The accredited hospital can provide prompt services	Srivastava & Goel [61]
	EX5	The accredited hospital informed healthcare conditions accurately.	Waraporn Sirithammanuku, BNS et al. [61]
Confirmation of Beliefs	CB1	The accredited h by PhilHealth was better than what I expected.	Qazi et al. [62]
	CB2	The accredited hospital provided more information than I expected.	Qazi et al. [62]
	CB3	I benefitted (less/more) from the accredited hospital by PhilHealth than I expected.	De Vreede et al. [63]
	CB4	I gained (less/more) from the accredited hospital by PhilHealth than I believe I would.	De Vreede et al. [63]
	CB5	I think the accredited hospital by PhilHealth met my expectations as a member of PhilHealth.	Qazi et al. [62]
Members Satisfaction	MS1	I am satisfied with the customer service of the accredited hospital by PhilHealth.	Collett Miles [64]
	MS2	I am pleased to be a member of PhilHealth.	Badran & Al-Haddad [65]
	MS3	I am pleased with the business operating system of the accredited hospital by PhilHealth.	Mas’adeh [66]
	MS4	I am pleased with the accredited hospital healthcare service.	Wu et al. [67]
	MS5	Overall, I think the accredited hospital by PhilHealth would have the best benefits and services for Filipino workers.	Reychav et al. [24]
Socio-Economic Factors	SF1	Are you satisfied with the present discounts offered by the PhilHealth	Dror et al. [68]
	SF2	Are you satisfied with the kind of service by hospitals, clinics, laboratories as accredited by PhilHealth	Panelo et al. [69]
	SF3	Are you satisfied with the present organizational chat of PhilHealth	Lau et al. [70]
	SF4	As to the number of accredited hospitals, clinics, and laboratories, is it serving the needs of the Filipinos when it comes to health service?	Dror et al. [68]
	SF5	What about your contribution? Is it compensatory with the service you get in the present when hospitalized (confined)	Panelo et al. [69]

**Table 3:** Activation function and optimizer.

Hidden Layer Activation Function	References
Sigmoid, Swish	Hung et al. [78]; Santosh et al. [88]; Sharma et al. [79]; Sim et al. [89]; Zoph & Le [80]
Sigmoid, Tanh	Elfwing et al. [81]; Kalinić et al. [83]; Liébana-Cabanillas et al. [87]
Elu	Clevert et al. [84]; Kiliçarslan & Celik [85]; Kim et al. [86]
Swish, Tanh, Elu, Sigmoid	Clevert et al. [84]; Jang & Park [82]; Sharma et al. [79]; Sim et al. [89]
**Output Layer Activation Function**	**References**
Sigmoid	Elfwing et al. [81]
Softmax	Yousefzadeh et al. [90]
Softmax, Sigmoid	Santosh et al. [88]; Saravanan and Sangeetha [91]; Wang et al. [39]
**Optimizer**	**References**
RMSProp	Bohmrah and Kaur, [95]; Xu et al. [99]; Yousefzadeh et al. [90]
SGD	Jena et al. [98]; Jena & Pradhan [97]
Adam	Kim and Choi [92]; Salem et al. [93]; Sommer et al. [94]

**Table 4:** Indicators statistical analysis.

Variable	Item	Mean	StD	Factor Loading	
				Initial	Final
Reliability	RL1	3.6740	0.89068	0.815	0.811
	RL2	3.3960	1.05328	0.560	0.663
	RL3	3.5780	1.03444	0.687	0.682
	RL4	3.6440	1.01554	0.679	0.680
Assurance	A1	3.5980	0.96657	0.760	-
	A2	3.6680	1.06209	0.632	-
	A3	3.5600	0.98608	0.631	-
	A4	3.5900	1.03152	0.772	-
Tangible	T1	3.5560	1.01633	0.602	-
	T2	3.6560	1.02360	0.679	-
	T3	3.6200	1.04776	0.689	-
	T4	3.5820	1.03413	0.679	-
	T5	3.6060	1.07754	0.729	-
Empathy	E1	3.5980	1.00719	0.664	-
	E2	3.5540	1.04560	0.733	-
	E3	3.5860	1.07746	0.657	-
	E4	3.6720	1.02690	0.643	-
Responsiveness	RS1	3.5320	1.02326	0.676	0.675
	RS2	3.6480	1.05372	0.683	0.692
	RS3	3.4980	1.02767	0.699	0.750
	RS4	3.6480	1.05181	0.640	0.702
	RS5	3.6140	1.06737	0.676	0.721
Perceived Performance	PP1	3.6480	0.94757	0.642	0.744
	PP2	3.4180	1.03606	0.533	0.733
	PP3	3.5660	1.09729	0.581	0.679
	PP4	3.5720	1.05596	0.602	0.692
	PP5	3.6120	1.04864	0.674	0.768
Expectation	EX1	3.5760	1.08193	0.579	0.657
	EX2	3.5740	1.05392	0.595	0.786
	EX3	3.5380	1.05393	0.574	0.667
	EX4	3.6260	1.06977	0.612	0.703
	EX5	3.5840	1.06075	0.622	0.715
Confirmation of Beliefs	CB1	3.5660	1.02163	0.556	0.744
	CB2	3.5800	1.05158	0.555	0.646
	CB3	3.5760	1.05568	0.630	0.719
	CB4	3.5820	1.05902	0.662	0.698
	CB5	3.6120	1.02936	0.639	0.723
Member Satisfaction	MS1	3.6160	1.04438	0.510	0.717
	MS2	3.7380	1.00766	0.541	0.723
	MS3	3.5820	1.06844	0.649	0.698
	MS4	3.6880	1.03188	0.523	0.688
	MS5	3.6320	1.09131	0.595	0.733
Socio-Economic Factor	SF1	3.6140	1.00948	0.609	0.609
	SF2	3.5220	1.12786	0.649	0.648
	SF3	3.4880	1.14564	0.723	0.723
	SF4	3.5720	1.16771	0.781	0.781
	SF5	3.4660	1.21482	0.775	0.775

**Table 5:** Model Fit.

Goodness of Fit Measures of SEM	Parameter Estimates	Minimum Cut-Off	Suggested by
Incremental Fit Index (IFI)	0.863	>0.80	Gefen et al. [100]
Tucker Lewis Index (TLI)	0.848	>0.80	Gefen et al. [100]
Comparative Fit Index (CFI)	0.862	>0.80	Gefen et al. [100]
Goodness of Fit Index (GFI)	0.831	>0.80	Gefen et al. [100]
Adjusted Goodness of Fit Index (AGFI)	0.803	>0.80	Gefen et al. [100]
Root Mean Square Error (RMSEA)	0.068	<0.07	Steiger [101]

**Table 6:** Composite Reliability and Validity.

Factor	Cronbach’s α	Composite Reliability (CR)	Average Variance Extracted (AVE)
Reliability	0.782	0.506	0.803
Responsiveness	0.816	0.502	0.834
Socio-Economic Factor	0.834	0.505	0.835
Expectation	0.822	0.500	0.833
Confirmation of Beliefs	0.816	0.501	0.833
Perceived Performance	0.817	0.524	0.846
Member Satisfaction	0.791	0.507	0.837

**Table 7:** Direct, Indirect, and Total Effects.

No	Variable	Direct Effect	*p*-Value	Indirect Effect	*p*-Value	Total Effect	*p-*Value
1	RS → EX	0.750	0.012	-	-	0.750	0.012
2	RS → PP	0.760	0.008	-	-	0.760	0.008
3	RL → EX	0.542	0.006	-	-	0.542	0.006
4	RL → PP	0.665	0.007	-	-	0.665	0.007
5	SF → EX	0.218	0.008	-	-	0.218	0.008
6	EX → CB	0.987	0.004	-	-	0.987	0.004
7	PP → CB	−0.657	0.028	-	-	−0.657	0.028
8	CB → MS	0.905	0.028	-	-	0.905	0.028
9	RS → CB	-	-	0.690	0.007	0.690	0.007
10	RS → MS	-	-	0.707	0.009	0.707	0.009
11	RL → CB	-	-	0.422	0.019	0.422	0.019
12	RL → MS	-	-	0.432	0.020	0.432	0.020
13	SF → CB	-	-	0.346	0.003	0.346	0.003
14	SF → MS	-	-	0.354	0.005	0.354	0.005
15	EX → MS	-	-	0.925	0.004	0.925	0.004
16	PP → MS	-	-	−0.673	0.029	−0.673	0.029

**Table 8:** Summary of Initial DLNN Run.

Latent	Nodes	Activation (H-Layer)	Activation (O-Layer)	Optimizer	Average Training	StDev	Average Testing	StDev
RL	100	Tanh	Softmax	Adam	47.81	8.459	80.50	4.403
A	90	Swish	Softmax	Adam	52.50	4.795	80.70	3.335
T	100	Tanh	Softmax	Adam	48.024	5.878	81.50	3.749
E	100	Tanh	Softmax	Adam	48.464	6.598	81.80	3.155
RS	100	Tanh	Sigmoid	Adam	44.889	9.235	81.60	5.461
PP	90	Tanh	Softmax	Adam	50.765	8.895	80.70	3.129
**EX**	**90**	**Tanh**	**Sigmoid**	**Adam**	**47.053**	**3.491**	**85.67**	**2.946**
CB	100	Tanh	Softmax	Adam	47.905	8.271	81.50	4.552
SF	90	Tanh	Softmax	Adam	51.039	10.475	80.40	5.641

Note: Bold represents the initial results.

## Data Availability

The data presented in this study are available on request from the corresponding author.
